# Filling the gaps in the research about second primary malignancies after bladder cancer: Focus on race and histology

**DOI:** 10.3389/fpubh.2022.1036722

**Published:** 2022-11-17

**Authors:** Belaydi Othmane, Zhenglin Yi, Chunyu Zhang, Jinbo Chen, Xiongbing Zu, Benyi Fan

**Affiliations:** ^1^Department of Urology, Xiangya Hospital, Central South University, Changsha, China; ^2^National Clinical Research Center for Geriatric Disorders, Xiangya Hospital, Central South University, Changsha, China

**Keywords:** second primary malignancies, bladder cancer, SEER database, histology, race

## Abstract

**Purpose:**

Previous research has shown that bladder cancer has one of the highest incidences of developing a second primary malignancy. So, we designed this study to further examine this risk in light of race and histology.

**Patients and methods:**

Using the surveillance, epidemiology, and end results (SEER) 18 registry, we retrospectively screened patients who had been diagnosed with bladder cancer between 2000 and 2018. We then tracked these survivors until a second primary cancer diagnosis, the conclusion of the trial, or their deaths. In addition to doing a competing risk analysis, we derived standardized incidence ratios (SIRs) and incidence rate ratios (IRRs) for SPMs by race and histology.

**Results:**

A total of 162,335 patients with bladder cancer were included, and during follow-ups, a second primary cancer diagnosis was made in 31,746 of these patients. When the data were stratified by race, SIRs and IRRs for SPMs showed a significant difference: Asian/Pacific Islanders (APIs) had a more pronounced increase in SPMs (SIR: 2.15; *p* 0.05) than White and Black individuals who had an SIRs of 1.69 and 1.94, respectively; *p* 0.05. In terms of histology, the epithelial type was associated with an increase in SPMs across all three races, but more so in APIs (IRR: 3.51; 95% CI: 2.11–5.85; *p* 0.001).

**Conclusion:**

We found that race had an impact on both the type and risk of SPMs. Additionally, the likelihood of an SPM increases with the length of time between the two malignancies and the stage of the index malignancy.

## Introduction

Bladder cancer is the sixth most common malignancy worldwide (1) and the 13th leading cause of cancer death globally, with an estimated death toll of 200,000 a year (2). Bladder cancer is around four times more common in men than women and is a disease of the elderly, with the majority of this type of patients with cancer being over 65 years (3). Studies have shown that there is a significantly increased risk of second primary malignancies in adult patients with bladder cancer compared to the general population (4). Risk factors for bladder cancer can include smoking, male sex, older age, genetic susceptibility, and exposure to certain chemicals (5), which can have a linkage to other types of cancer and thereby contribute to a second primary carcinoma among bladder cancer survivors.

With the growing population of bladder cancer survivors due to an increase in survival that has been ascribed to a variety of variables, including earlier detection, advancements in cancer-specific therapy, and supportive care (6). One area that is not so highly explored is the racial and histological variations in the likelihood of developing a second primary after a bladder cancer diagnosis. Studying a population-based, racially diverse sample of bladder cancer survivors would help determine the relationships between race, histology, and the risks of developing an SPM. Indeed, understanding racial and histological differences in these patients will improve our understanding of the disparities in second primary incidence/risk and help us create better patient care strategies such as aiding in clinical decision-making about monitoring and following patients diagnosed with bladder cancer (BCa). Also, examining the risks of subsequent malignancies following bladder cancer and the variation in these risks by race and ethnicity may help to support hypotheses regarding genetic and lifestyle factors impacting bladder cancer risk because initial and subsequent cancers can share risk factors.

There are an increasing number of studies examining the risk factors for SPC in many types of tumors, such as prostate (7) and lung cancers (8). However, these studies analyzed second primaries-related variables using logistic regressions or Cox proportional hazard regressions, excluding death as a competing event for the occurrence of SPMs. Moreover, the influence of race and histological variants, as well as other variants, on patients with BCa when it comes to second malignancies is still not completely known. And to the best of our knowledge, no study has been conducted to measure the differences between these variables on bladder cancer SPMs. It is, thus, required to investigate and identify relevant variables, namely risk factors associated with second primaries in patients with bladder cancer. Therefore, this research is the first systematic retrospective study to examine the risk and distribution of SPMs in light of racial/histological variants following bladder cancer diagnoses by utilizing a large sample of such cases acquired from the surveillance, epidemiology, and end results (SEER) Program.

## Patients and methods

We extracted the data from the SEER 18 registry database by using the SEER^*^Stat 8.3.9.1 program. The SEER database (https://seer.cancer.gov) represents 28% of the U.S. population in terms of demographics and cancer incidence and collects data that include race, primary tumor site, follow-up time for new malignancies, stage, and grade. The superiority of the SEER database is also apparent in its capability to recognize a second primary and not misdiagnose it because it follows a series of coding rules that considers site, histology, mucosal involvement in anastomotic lesions, and follow-up time.

Our study population acquired from it included patients diagnosed with first primary bladder cancer at ages 20–84 between 2000 and 2018, as it was submitted to one of 18 SEER cancer registries in November 2020. Patients were disqualified if their data came from autopsy or death certificates only. We also excluded non-melanoma skin cancers as they are not routinely collected by the database. Other exclusion criteria included patients with missing values on important covariates such as race, stage, and grade. And in order to analyze specific second malignancies, the diagnosis of another second primary was a censoring event.

The information collected was regarding the age at diagnosis, gender, race, pathological type, degree of pathological differentiation, year of diagnosis, primary lesion site, whether radiotherapy and chemotherapy were received, survival time, and survival status. Patients were separated into three age groups as follows: 35–59, 60–69, and 70–84 years old, while race into White, Black, and Asian/Pacific Islander individuals. The histological type was categorized by the database into transitional cell carcinoma, squamous cell carcinoma, epithelial neoplasm, adenocarcinoma, and “others,” which was an aggregation of other types of histological variants that did not belong to the previous four and were statistically insignificant individually. Authorization was obtained through the SEER website, and data were retrieved from the SEER database, with no additional ethical approval being required.

The follow-time started 2 months after a bladder cancer diagnosis in order to reduce the likelihood of incidental second malignancies inclusions due to increased diagnostic workup and surveillance. This follow-up time continued until the diagnosis of a second primary, and it stopped in the case of 85 years and older (exclusion for patients in such old age was because of concerns about under-reporting new primaries in this age group), death, or December 2018.

### Analysis

SIR is the ratio of the observed to the expected number of events, with expected values being estimated using race-/histology-specific cancer incidence rates in the general population of the 18 SEER database. This standardized incidence ratio (SIR) was computed and analyzed using the SEER^*^Stat 8.3.9.1. Furthermore, the corresponding 95% confidence level (CI) was reported. Other analyses were done using the StataMP 17. Analyses were stratified by mutually exclusive categories of histology and race. For histological variants categories: transitional cell carcinoma, squamous cell carcinoma, epithelial carcinoma, adenocarcinoma, and others; for race: White, Black, and Asian/Pacific Islander individuals. Assessing SIRs by latency was done to examine the extent of medical surveillance bias in case it leads to more cancer diagnoses in the general population because they were simply under more close medical scrutiny, which tends to be in the 1st years after diagnosis. Other analyses included incidence rate ratios (incidence rate in the exposed group/incidence rate in the unexposed group using multilevel Poisson models) with 95% confidence intervals (95% CI) of SPM within race/histology adjusted by multiple variables, such as sex, race, age, and calendar year. Because the SIR has a limitation in that it uses the general population as a comparison group making this a diagnostic bias [cancer survivors receive more screening, leading to an increased likelihood of diagnosing a second malignancy than the general population (9)], we ran the Poisson regression on the different variables of BCa survivors to examine the relative risk of developing site-specific cancers for these patients which would help paint a broader picture on the results at hand. The competing risk was computed using Fine and Gray's model to give lower weight to patients with the competing event (death was the competing event). This would help us not to overestimate the risk of SPMs since the Cox proportional hazard model would have treated every patient who did not have an event at the end of the follow-up as censored. This is problematic because bladder cancer survivors might either die during the follow-up or remain alive at the end of it, but the Cox analysis considers both of these scenarios as equal even when these BCa survivors who died had no probability of developing an SPM, which would make the assumption of independence unfulfilled in this case, resulting in inaccurate survival rates (10). In addition to this, a subdistribution cumulative incidence function was plotted.

## Results

A total of 162,335 patients diagnosed with bladder cancer between 2000 and 2018 were screened from the SEER database, among which 31,746 patients had a subsequent cancer diagnosis starting after a 2-month period (Table 1). The largest sample according to race was Whites with 90% (*n* = 145,758), followed by Black individuals and Asians or Pacific Islanders (APIs) with 5.6% and 4.1%, respectively. Closer to two decades of follow-up, these cases ended up developing second primary cancers in a significant percentage among the different races, 91.7% (*n* = 29,099) among White people, 5% (*n* = 1,586) among Black individuals, and 3.3% (*n* = 1,061) among API people. IRRs (Table 2) had a similar trend but due to different reasoning, as mentioned in the discussion. Overall, bladder cancer survivors had a considerably higher risk of second primaries compared to the general population, with an SIR of 1.72 (95% CI: 1.70–1.74; *p* < 0.05). But when it came to stratifying the results by the different races, the SIRs varied in the manner that APIs had a more pronounced increase (SIR: 2.15; *p* < 0.05) than Whites and Black individuals with an SIR of 1.69 and 1.94, respectively; *p* < 0.05 (Table 3).

**Table 1:** Bladder cancer cases by race.

		**Total**		**White individuals**		**Black individuals**		**Asian/Pacific Islanders**	
		***n***	**Percent**	***n***	**Percent**	***n***	**Percent**	***n***	**Percent**
Total		161,335	100.00%	145,758	90.30%	8,975	5.60%	6,602	4.10%
Sex	Male	123,982	76.80%	112,809	77.40%	6,077	67.70%	5,096	77.20%
	Female	37,353	23.20%	32,949	22.60%	2,898	32.30%	1,506	22.80%
Age	35–59	35,738	22.20%	31,636	21.70%	2,642	29.40%	1,460	22.10%
	60–69	48,774	30.20%	44,023	30.20%	2,880	32.10%	1,871	28.30%
	70–84	76,823	47.60%	70,099	48.10%	3,453	38.50%	3,271	49.50%
Year	2000–2004	46,919	29.10%	43,059	29.50%	2,322	25.90%	1,538	23.30%
	2005–2009	46,251	28.70%	41,950	28.80%	2,440	27.20%	1,861	28.20%
	2010–2014	39,941	24.80%	35,710	24.50%	2,474	27.60%	1,757	26.60%
	2015–2018	28,224	17.50%	25,039	17.20%	1,739	19.40%	1,446	21.90%
Histology	Transitional	155,631	96.50%	140,941	96.70%	8,356	93.10%	6,334	95.90%
	Squamous	2,489	1.50%	2,164	1.50%	239	2.70%	86	1.30%
	Epithelial	1,245	0.80%	1,080	0.70%	102	1.10%	63	1.00%
	Adenocarcinoma	1,109	0.70%	890	0.60%	146	1.60%	73	1.10%
	Others	861	0.50%	683	0.50%	132	1.50%	46	0.70%
Stage	*In situ*	81,973	50.80%	75,314	51.70%	3,540	39.40%	3,119	47.20%
	Localized	59,875	37.10%	53,577	36.80%	3,711	41.30%	2,587	39.20%
	Regional	13,847	8.60%	12,062	8.30%	1,133	12.60%	652	9.90%
	Distant	5,640	3.50%	4,805	3.30%	591	6.60%	244	3.70%
Grade	I	23,542	14.60%	21,673	14.90%	1,162	12.90%	707	10.70%
	II	52,670	32.60%	48,129	33.00%	2,517	28.00%	2,024	30.70%
	III	33,980	21.10%	30,491	20.90%	2,171	24.20%	1,318	20.00%
	IV	51,143	31.70%	45,465	31.20%	3,125	34.80%	2,553	38.70%
Radiation	No/unknown	154,153	95.50%	139,574	95.80%	8,294	92.40%	6,285	95.20%
	Yes	7,182	4.50%	6,184	4.20%	681	7.60%	317	4.80%
Chemotherapy	No/unknown	127,395	79.00%	115,562	79.30%	6,846	76.30%	4,987	75.50%
	Yes	33,940	21.00%	30,196	20.70%	2,129	23.70%	1,615	24.50%
Follow-up	Event	31,746	19.70%	29,099	20.00%	1,586	17.70%	1,061	16.10%
	Censored	72,359	44.90%	65,208	44.70%	3,562	39.70%	3,589	54.40%
	Death	57,230	35.50%	51,451	35.30%	3,827	42.60%	1,952	29.60%

**Table 2:** SPM cases by race using the IRR, with 95% CI; only *p* > 0.001 are mentioned.

**Variable**		**White individuals**		**Black individuals**		**Asian/Pacific islanders**	
		***n***	**IRR (95%CI)**	***n***	**IRR (95%CI)**	***n***	**IRR (95%CI)**
Sex	Male	24,330	ref	1,213	ref	864	ref
	Female	4,769	0.89 (0.86–0.92)	373	1.4 (1.24–1.57)	197	1.04 (0.89–1.21) *P* = 0.654
Age	35–59	4,927	ref	389	ref	146	ref
	60–69	9,674	1.21 (1.17–1.25)	576	0.91 (0.80–1.04) *P =* 0.156	316	1.33 (1.09–1.62) *P =* 0.004
	70–84	14,498	1.53 (1.48–1.58)	621	0.83 (0.73–0.94) *P =* 0.004	599	2.13 (1.78–2.55)
Year	2000–2004	11,057	ref	545	ref	327	ref
	2005–2009	9,547	1.25 (1.22–1.28)	529	1.40 (1.25–1.58)	382	1.69 (1.46–1.96)
	2010–2014	6,048	1.99 (1.93–2.05)	355	2.37 (2.07–2.71)	232	2.58 (2.18–3.05)
	2015–2018	2,447	4.35 (4.16–4.54)	157	5.66 (4.74–6.76)	120	7.21 (5.85–8.89)
Histology	Transitional	28,416	ref	1513	ref	1018	ref
	Squamous	310	1.02 (0.92–1.12) *P =* 0.673	21	1.30 (0.85–2.00) *P =* 0.228	11	1.01 (0.56–1.84) *P =* 0.962
	Epithelial	166	1.39 (1.20–1.62)	18	2.84 (1.78–4.52)	15	3.51 (2.11–5.85)
	Adenomas	126	1.05 (0.88–1.25) *P =* 0.579	21	1.05 (0.89–1.25)	13	3.03 (1.75–5.23)
	Others	81	1.35 (1.08–1.68) *P =* 0.007	13	3.29 (2.14–5.06)	4	1.86 (0.70–4.96) *P =* 0.216
Stage	*In situ*	16,521	ref	804	ref	570	ref
	Localized	10,135	1.14 (1.12–1.17)	589	1.37 (1.23–1.52)	393	1.29 (1.13–1.46)
	Regional	2,207	1.83 (1.75–1.91)	162	2.76 (2.33–3.26)	80	1.92 (1.52–2.43)
	Distant	236	2.31 (2.03–2.62)	31	6.23 (4.35–8.92)	18	5.10 (3.19–8.16)
Grade	I	4,468	ref	228	ref	105	ref
	II	9,746	1.02 (1.05–0.98) *P =* 0.407	487	0.99 (0.85–1.16) *P =* 0.940	342	1.52 (1.22–1.89)
	III	6,418	1.17 (1.13–1.22)	370	1.32 (1.12–1.56) *P =* 0.001	239	1.86 (1.48–2.34)
	IV	8,467	1.50 (1.45–1.56)	501	1.74 (1.49–2.04)	375	2.83 (2.28–3.51)
Radiation	No/unknown	28,528	ref	1,536	ref	1,033	ref
	Yes	571	1.53 (1.41–1.66)	50	2.49 (1.88–3.29)	28	2.07 (1.42–3.01)
Chemotherapy	No/unknown	24,170	ref	1,317	ref	848	ref
	Yes	4,929	1.42 (1.38–1.47)	269	1.42 (1.25–1.62)	213	1.75 (1.51–2.04)
Latency	1–5 years	10,871	ref	667	ref	414	ref
	>5 years	18,228	0.26 (0.25–0.27)	889	0.20 (0.18–0.22)	647	0.24 (0.21–0.27)

**Table 3:** SPM cases by race using the SIR, with 95% CI; ^*^: *p* > 0.05.

		**White individuals**	**Black individuals**	**Asian/Pacific islanders**
		**SIR (95%CI)**	**SIR (95%CI)**	**SIR (95%CI)**
Total		1.69 (1.67–1.71)	1.94 (1.85–2.04)	2.15 (2.02–2.29)
Sex	Male	1.72 (1.7–1.75)	1.91 (1.8–2.02)	2.13 (1.99–2.27)
	Female	1.54 (1.5–1.59)	2.06 (1.86–2.28)	2.28 (1.97–2.63)
Age	35–59	1.95 (1.9–2.01)	2.03 (1.83–2.25)	2.22 (1.87–2.61)
	60–69	1.74 (1.7–1.77)	1.91 (1.76–2.08)	2.26 (2.01–2.53)
	70–84	1.59 (1.56–1.62)	1.92 (1.77–2.07)	2.09 (1.92–2.26)
Year	2000–2004	1.44 (1.41–1.47)	1.64 (1.51–1.79)	1.62 (1.45–1.81)
	2005–2009	1.66 (1.62–1.69)	1.95 (1.79–2.13)	2.27 (2.05–2.51)
	2010–2014	2.07 (2.02–2.13)	2.19 (1.96–2.43)	2.47 (2.16–2.81)
	2015–2018	2.83 (2.71–2.94)	3.04 (2.58–3.56)	3.99 (3.31–4.77)
Histology	Transitional	1.68 (1.66–1.7)	1.93 (1.83–2.03)	2.12 (1.99–2.26)
	Squamous	1.7 (1.52–1.91)	1.72 (1.06–2.63)	1.8* (0.82–3.41)
	Epithelial	2.75 (2.34–3.22)	3.93 (2.33–6.21)	4.34 (2.37–7.29)
	Adenocarcinomas	2.14 (1.78–2.56)	2.09 (1.29–3.19)	5.00 (2.66–8.55)
	Others	2.32 (1.83–2.89)	2.02 (1.07–3.45)	3.17* (0.65–9.28)
Stage	*In situ*	1.59 (1.56–1.61)	1.91 (1.78–2.05)	2.12 (1.95–2.3)
	Localized	1.68 (1.65–1.71)	1.75 (1.61–1.9)	2.04 (1.84–2.25)
	Regional	3.32 (3.19–3.47)	3.24 (2.76–3.78)	2.99 (2.36–3.75)
	Distant	2.61 (2.28–2.97)	3.57 (2.41–5.1)	5.25 (3.11–8.3)
Grade	I	1.37 (1.33–1.41)	1.46 (1.27–1.66)	1.59 (1.3–1.93)
	II	1.46 (1.43–1.48)	1.63 (1.49–1.79)	1.90 (1.59–1.3)
	III	1.82 (1.77–1.86)	2.12 (1.91–2.35)	2.26 (1.98–2.57)
	IV	2.29 (2.24–2.34)	2.69 (2.45–2.93)	2.67 (2.4–2.96)
Radiate	No/unknown	1.69 (1.67–1.71)	1.95 (1.86–2.05)	2.15 (2.02–2.29)
	Yes	1.65 (1.51–1.79)	1.67 (1.23–2.21)	2.28 (1.52–3.3)
Chemotherapy	No/unknown	1.61 (1.59–1.63)	1.89 (1.79–2)	2.06 (1.92–2.21)
	Yes	2.22 (2.16–2.29)	2.23 (1.97–2.51)	2.60 (2.26–2.98)
Latency	1–5 years	3.83 (3.76–3.9)	4.15 (3.84–4.47)	4.82 (4.36–5.32)
	>5 years	1.27 (1.25–1.29)	1.38 (1.29–1.47)	1.59 (1.47–1.72)

### Results according to the IRRs and SIRs

To determine the equality or disparity of different variables in developing a second primary, we examined the heterogeneity of IRRs stratified by the specific factors mentioned in Table 1 across the three ethnic groups (Table 2). IRRs were significantly different by sex in both White and Black individuals' cases but not so in API people; being a woman increased the association of developing a second primary more apparently among Black individuals (IRR: 1.40, CI: 1.24–1.57). For age, White and API cases varied significantly, *p* < 0.005, but not for Black individuals in the 60–69 years subgroup as *p* = 0.156. For histology, there were also non-significant differences between the different histological variants, except for the epithelial subtype across all the three races and “others” among the White and Black individuals' cases. The epithelial subtype was associated with all three races but more so among API cases (IRR: 3.51 CI: 2.11–5.85). Receiving radiation and chemotherapy also establishes a close link for developing an SPC among the different races: radiation variable IRRs for Whites (IRRs: 1.53, 95% CI: 1.41–1.66), Black individuals (IRRs: 2.49, 95% CI: 1.88–3.29), and APIs (IRRs: 2.07, 95% CI: 1.42–3.01) increased with the receiving groups. This was similar for chemotherapy with IRRs of 1.42, 1.24, and 1.75, respectively; *p* < 0.001.

SIRs correlated with most of the IRRs (Table 3), although since SIRs are a comparison to the general population, this would give us a more extensive insight on which variable had a more increased risk of developing second primaries, so it would be beneficial to go over it. For the histological variants, the epithelial subtype ranked first with its association with SPMs compared to the general population, followed by “others” and then by adenocarcinomas for Whites (SIR: 2.75, 95% CI: 2.34–3.22; 2.32, 95% CI: 1.83–2.89; and 2.75, 95% CI: 2.34–3.22, respectively). For Black individuals' cases, epithelial subtype also ranked first but adenocarcinomas preceded “others” (SIR: 3.93, 95% CI: 2.33–6.21; 2.09, 95% 1.29–3.19; and 2.02, 95% CI: 1.07–3.4, respectively); for APIs, adenocarcinomas had the highest SIR (SIR: 5.00, 95% CI: 2.66–8.55) followed by the epithelial subtype (SIR: 4.34, 95% CI: 2.37–7.29). Moving on to cancer stages, the highest SIR was found to be within the regional type for Whites (SIR: 3.32, 95% CI: 3.19–3.47) and distant type for Black individuals (3.57, 95% CI: 2.41–5.1) and APIs (5.25, 95% CI: 3.11–8.3). Turning to latency, we found out that during the first 5 years, a higher SIR is exhibited among all races compared to a period beyond 5 years; API cases (SIR: 4.82, 95% CI: 4.36–5.32) had the first rank in terms of SIRs, followed with Black individuals (SIR: 4.15, 95% CI: 3.84–4.47), and then Whites (3.83, 95% CI: 3.76–3.9) during the 1–5 years period group. All the SIRs' figures commented on here had a *p*-value of < 0.05.

### Incidence of SPMs by anatomical sites

**Figure 1:**
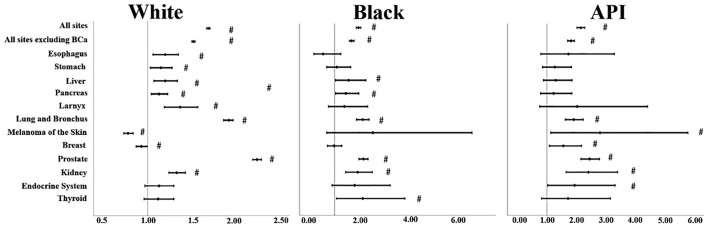
SIRs for second primaries at various anatomical sites based on the different races. “#” signified an SIR of *p* < 0.05.

For sites, some results of SIRs were somehow insignificant when it came to Black and API individuals' cases due to limited analytical power (*p* > 0.05) (Supplementary Table 1); so, we chose the malignancies mentioned in Figure 1 to comment on. SIRs varied significantly by race. For “all sites,” APIs had the highest SIR, followed by Black and White individuals' cases. This was consistent also when we excluded bladder cancer from the analysis, although that exclusion lowered the SIR for each race: Whites (SIR: 1.52, 95% CI: 1.5–1.54), Black individuals (SIR: 1.67, 95% CI: 1.58–1.77; *p* < 0.05), and APIs (SIR: 1.82, 95% CI: 1.7–1.94; *p* < 0.05). Lung and bronchus malignancies were on the rise consistently in all three races: Black individuals (SIR: 2.11, 95% CI: 1.86–2.37; *p* < 0.05) followed by Whites (SIR: 1.92, 95% CI: 1.86–1.97) and APIs (SIR: 1.91, 95% CI: 1.62–2.24; *p* < 0.05). In its footsteps, prostate and kidney cancers SIRs also were increased with APIs (SIR for the prostate: 2.45, 95% CI: 2.15–2.79 vs. kidney 2.41, 95% CI: 1.65–3.4; *p* < 0.05) taking the lead for both types but Black individuals' cases took the lead in kidney malignancies (SIR: 1.91, 95% CI: 1.43–2.49) compared to Whites (SIR: 1.33, 95% CI: 1.24–1.43), while this was reversed for prostate cancer since Whites (2.24, 95% CI: 2.19–2.29; *p* < 0.05) had the second-highest SIR. For digestive cancers such as esophagus and stomach cancers, although the lower confidence limit was closer to 1 for both of these cancers, there was an increase in the incidence rate of Whites (SIR for esophagus: 1.20, 95% CI: 1.06–1.35 and for stomach: 1.15, 95% CI: 1.03–1.28; *p* < 0.05) compared to the general population, but this was blurred out for Black and API individuals' cases as their *p*-values were > 0.05. We noticed similar results in larynx cancer for Whites (SIR: 1.37, 95% CI: 1.19–1.57; *p* < 0.05). Liver and pancreas SIRs increased for both Whites (SIR for liver: 1.20, 95% CI: 1.07–1.34 vs. pancreas: 1.13, 95% CI: 1.04–1.23; *p* < 0.05) and Black individuals (SIR for liver: 1.55, 95% CI: 1.03–2.24 vs. pancreas: 1.44, 95% CI: 1.03–1.96; *p* < 0.05); however, it is good to keep in mind that most of their lower confidence limit was closer to 1. On the other hand, APIs had a non-significant SIR; *p* > 0.05.

### Sub-distribution hazard ratio and the cumulative incidence of SPMs

**Figure 2:**
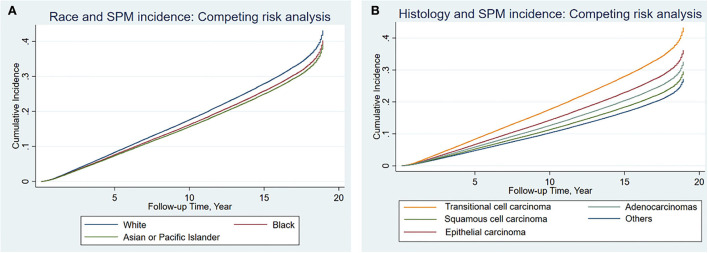
Cumulative incidence of second primary malignancies among the different races **(A)** and histological variants **(B)** in bladder cancer based on the Fine-Gray method.

Results from the competing risk analysis might be different from that of SIR since, in this analysis, developing a second primary cancer is the primary outcome of concern; therefore, death before an SPM is counted as a competing risk. The sub-distribution hazard ratio of developing SPMs was associated significantly with White cases than with Black individuals (SHR: 0.91, 95% CI: 0.87–0.96). Although such a difference can be considered to be of such a low impact despite the low *p*-value since the upper 95% CI: is closer to 1. Black individuals were then followed by APIs (SHR: 0.88, 95% CI: 0.83–0.93) (Table 4), and the CIF confirmed the high risk associated with White cases than Black individuals and APIs and was more significant with 10 years follow-up onward (Figure 2). For histological variants (Table 5), SPMs were associated more with transitional carcinomas, then followed up by epithelial neoplasms (0.79, 95% CI: 0.69–0.91); all *p*-values were < 0.001.

**Table 4:** Sub-distribution hazard ratio of developing second primaries after a bladder cancer diagnosis among the different races; White cases are the Ref.

	**SHR**	**95% conf. interval**	***P*-value**
Black	0.91	0.87–0.96	< 0.001
API	0.88	0.83–0.93	< 0.001

**Table 5:** Sub-distribution hazard ratio of developing second primaries after a bladder cancer diagnosis among the different histological variants; transitional carcinomas are the Ref.

	**SHR**	**95% conf. interval**	***P*-value**
Squamous cell carcinoma	0.62	0.56–0.69	< 0.001
Epithelial neoplasm, NOS	0.79	0.69–0.91	< 0.001
Adenocarcinoma	0.69	0.59–0.81	< 0.001
Others	0.56	0.46–0.68	< 0.001

## Discussion

Compared to the general population, patients with bladder cancer had a greater overall risk of SPMs with an SIR of 1.72, and around 19% percent of bladder cancer diagnosed survivors ended up developing an SPM, which is consistent with other studies (4). For example, one study by Nicholas Donin et al. found that bladder cancer survivors have 19% of developing a second primary cancer at 10 years of follow-up time and 34% at 20 years (11). Men (SIR: 1.72) were also at greater odds of developing an SPM compared to the general population than women in this instance (SIR: 1.54).

Similar to other studies (12, 13), our study also found that prostate cancer was the most often encountered second primary malignancy in patients with bladder cancer, followed by lung cancer, colon cancer, kidney and renal pelvis cancer, and breast cancer. Interestingly, these tumors are also the most common types of cancer in general as a single malignancy. Explanations for the increased incidence of particular tumors as SPMs, such as prostate cancer, may be explained by the fact that patients are susceptible to both malignancies by sharing a similar carcinogenic pathway, such as DNA repair and N-acetyltransferase 2 (NAT2) (14, 15), which could explain the link between these two cancers. Other explanations for the rise in prostate cancer diagnoses might stem from both cancers becoming more common in middle-aged patients. A relationship between infection and the bladder and prostate tumors has also recently been established in recent studies (16, 17).

We also confirmed with this large population-based investigation that the risk of acquiring second malignancies differed according to patients' race and the histological variants from the first bladder cancer diagnosis. Our findings imply that the overall risk reduction does not apply to all race groups; as we saw, in comparison to the general population, patients with API bladder cancer had a considerably elevated risk of all subsequent malignancies (SIR: 2.15). Site-specific risk of SPC also differed between White, API, and Black individuals. API bladder cancer cases had a significantly increased risk of prostate, breast, and endocrine cancers compared to other races. For Black individuals' cases, they had more increased risk of lung and bronchus malignancies SPMs, while Whites took the lead in digestive cancers. This pattern is more likely to be explained by socioeconomic and lifestyle factors, but genetic differences between White and Black individuals could also potentially play a role in the disease's molecular genesis and pathogenesis (18). These variables, for example, could affect how the host reacts to carcinogens and other environmental elements. As a result, if one race has disproportionately more extensive exposure to carcinogens or has a different metabolism of these carcinogens than the other races, then there might be differences in their risks of second primaries.

To add to this interaction, we can look at the SIR of lung cancer. We found that lung and bronchus cancers were on the increase across the board in all the three groups, but Black individuals (SIR: 2.11, 95% CI: 1.86–2.37; *p* < 0.05) had the highest incidence ratio. Giving an account of why lung cancer is raised in all three races would be to mention that the increased risk of developing these tumors in patients with a history of bladder cancer may also be due to common etiologies. This was shown in a study (19) that demonstrated an increased risk of lung cancer and kidney cancer in patients initially diagnosed with bladder cancer, and they ascribed this risk to smoking, which was also consistent with our study. In general, cigarette smoking is widely recognized as the most significant underlying cause of various cancers (20, 21). Also, cigarette smoking is a significant risk factor for bladder cancer (21) and a well-established risk factor for lung and bronchus cancer. In addition, it is interesting to note that Black individuals' cases in our data had the highest rate of lung cancer as an SPM, and this might be explained by looking at data from the National Survey on Drug Use and Health (NSDUH), in which menthol cigarette use is more prevalent among Black individuals, with Black people having almost 25 times the likelihood of smoking it compared to White people. Furthermore, there has been evidence that marketing methods and product development were purposely concentrated on Black individuals' neighborhoods in the USA, which would only increase the rate of smoking in this demographic and make it hard for people to stop smoking (22–24). For a biological reason behind such increased risk in Black individuals compared to other races, one study did not find any significant differences in cancer-related mutations between the different races regarding lung cancer (25). Another study, however, found that RB1 mutations were higher in African Americans than in European Americans in patients with lung cancer. What is interesting is that the same RB1 is also mutated at a higher percentage in patients with bladder cancer, so there might be an association with the higher increase in both of those cancers specifically for Black individuals (26, 27). Besides this, more studies are still needed to confirm more clearly what type of mutations are responsible for the rise in lung cancer among Black patients, especially when they have lesser access to genetic testing and top research institutions (28), and according to the National Lung Cancer Screening Trial, only around 4% of their cohort were Black individuals compared to a majority of Whites. This absence of diversity hinders relevant findings and the application of the results to clinical settings (29).

Another thing that was distinguished in the SPMs according to race was that breast cancer incidence rates increased within APIs (SIR: 1.56, 95% CI: 1.08–2.18; *p* < 0.05) while decreased among Whites (SIR: breast: 0.93, 95% CI: 0.87–1; *p* < 0.05), with the caveat that the upper confidence limit is 1, so it is of marginal statistical significance. API is an aggregation of different subgroups from the different regions of Asia and the Pacific Islands. However, we can still speculate that this rise might be attributed to one subgroup over another, reflecting the increase in the entire category, especially when we have indications for this. The increase in breast cancer in API cases might be due to the genomic Arg72Pro substitution in the p53 protein and its association with bladder cancer in Asians. According to Marei et al. (30), mutations in the TP53 gene disturb the cell cycle, leading cells to lose control over cell proliferation, and resulting in the transfer of damaged DNA to their progeny, which eventually develops into malignant cells; around half of all human breast, colon, lung, liver, prostate, and bladder cancers have p53 mutations. One specific allele variation of the p53 is what is relevant here, especially when this allele (The Arg72Pro polymorphism variant of the p53 gene) was proved to have a significant impact on p53 biological activity as well as causing much more G1 arrest than the Arg72 form (31). In Asian patients, a significant association between p53 codon 72 polymorphism and bladder cancer risk is found in the additive model of the Arg72Pro with an OR of 1.72 and the dominant model with an OR of 1.27, but not in Whites, according to Zhili Yang et al. meta-analysis (32). Similar findings were present in this allele association with breast cancer. This was also confirmed by multiple studies where the carriers of the Arg72Pro allele were shown to have a higher risk of breast cancer in both the dominant and additive models compared to the wild type (wtTP53) (33, 34). This would follow that because a significant percentage of breast cancer have p53 mutations, it is reasonable to posit that patients with bladder cancer as an index cancer that is also associated with p53 mutation on the same allele would have an increased incidence of breast cancer as a second primary malignancy.

Esophagus cancers had an increased incidence in White cases (SIR for esophagus: 1.20, 95% CI: 1.06–1.35), with a lower confidence limit that was somehow closer to 1, while it was not statistically significant among the other races. This may be due to genetic reasons or an increase in medical surveillance, especially since Whites had a five times more chance of developing esophageal cancer than Black individuals (35). Whites also had a rise in gastric cancers (1.15, 95% CI: 1.03–1.28; *p* < 0.05), while the other ethnicities had a non-significant increase. This can only be explained in part by the prostate stem cell antigen (PSCA), which is responsible for gastric cancer and bladder cancer at the same time because it is responsible for these two types of cancer across different ethnicities (36, 37). This gene might also be linked to the high incidence of prostate cancer as an SPM in our results as PSCA is a glycosylphosphatidylinositol (GPI)-anchored cell surface protein expressed by prostate stem cells and is reported to be increased in the epithelium of various typical human tissues, including the urinary bladder, kidney, skin, esophagus, stomach, and placenta epithelium. PSCA has also been linked to various cancers besides gastric and prostate cancer, such as pancreatic cancer, according to a growing body of evidence (38).

In general, when it comes to race, according to the data we obtained from the SEER database, the highest rates of second primaries were found in Black individuals and API cases, while the lowest rates were found in White cases. Social and lifestyle factors are more likely to explain this trend, and there may also be genetic differences between Black and White people that play a role in the disease's molecular origin and pathogenesis (18). Examples of such variables include the host's reaction to particular carcinogens and environmental conditions, among other things. Other hypotheses that might explain the discrepancies include the fact that Whites had a lower rate of second primaries than Black individuals, which could be linked to their general lower cigarette usage, as we mentioned before, which might have ended up increasing the risk of Black individuals' cases toward having lung cancer as an SPM. However, cigarette smoking can also extend to other cancer, as cigarette smoking is widely accepted to be the most significant environmental risk factor for a variety of cancers (20).

For histology, although more confirmatory studies are still required, in particular, studies with available information on significant confounders like lifestyle factors, genetic susceptibility, and detailed treatment data, our analysis highlighted the importance of segmenting BCa according to histological subtypes in that the risk and distribution of SPMs vary according to the primary BCa histological subtype. Different studies have shown to certain degrees that the histological subtypes of bladder cancer guide treatment decisions because different subtypes have distinct degrees of susceptibility to the available therapies, resulting in differences in exposures to chemotherapy and radiation (39, 40). Receiving chemotherapy for some subtypes, for example, would affect healthy tissues within the radiation field, and then radiation-induced second tumors might develop. In addition, each histological subtype has unique characteristics, including higher mortality for histological variants such as squamous cell carcinoma and distinct metastatic potentials, which might upset the incidence of SPMs (41, 42). The patterns of SPMs following BCa would also have other overlapping mechanisms, such as common risk or preventative factors and genetic predispositions. Therefore, doctors should be aware that the risk of SPM development varies among patients with bladder cancer with different histological subtypes, which might result in better follow-up strategies.

Furthermore, the carcinogenic effects of radiotherapy or chemotherapy may have contributed to the increased risk in the latter period, especially in our study, where regardless of race, prior chemotherapy and radiation treatments were observed to be positively associated with the development of SPMs rather than acting as a preventative measure (the 2.49 IRR on Black individuals seemed particularly interesting); keeping in mind, that due to the small sample sizes used for this analysis the differences may have been amplified between the three ethnicities. To examine this further, we can look at radiation exposure, which is a well-established risk factor for cancer development (9) and is more likely to be used to treat more aggressive diseases. Typically, a person's risk of developing cancer due to radiation exposure grows over time (43), and recent studies have also added to this by showing that people, for example, who have had prostate cancer radiotherapy are more prone than those who have not to get SPMs of the bladder and colorectal area (44). These secondary bladder cancers can arise after a 5- to 15-year follow-up time, and they can be more aggressive or progressed at the time of diagnosis. Although there are very few studies tackling this subject of determining the specific risk of secondary cancer after BCa radiotherapy, one study found that secondary cancers following bladder cancer are more common in the gastrointestinal system, including the intestine, colorectal area, and genital systems than in patients who did not receive radiation therapy (45). This leads us to conclude that their findings are analogous to prostate cancer research findings (44). However, there was a flaw in their study: the lack of specific information, such as the type of radiation therapy utilized and the amount of radiation given.

In terms of latency, we found that all races had the highest risk during the first 5 years, with APIs in the first place, followed by Black individuals and then White cases. The high incidence in these 1st years might be due to the diagnostic workup performed on the index cancer. Consistent with our results, one study found that the risk of second prostate cancer (PCa) and kidney malignancies did not differ significantly depending on the length of follow-up. After 6 months of follow-up, the incidence of prostate cancer had settled at a rate that was three to four times higher than what was anticipated, and second kidney cancers had similar results, with slight variation (46). With a 5-year follow-up, the high incidence rate of three to four times greater in developing these two types of cancer remained. Therefore, it is somehow useful if practitioners would conduct regular standard tests for early diagnosis of these 2-second primary malignancies, including PSA test and annual ultrasounds of the kidneys. For second cancers of the lung prevention, on the other hand, it is better to follow it up during the second to the 5th year, especially for Black individuals' cases, as they have lung cancer as the highest occurring SPM. These lung cancer follow-ups, which are mainly done by a chest radiograph, are also a recommendation for bladder cancer survivors in general after a cystectomy (47).

For the sub-distribution hazard ratio mentioned in Table 4, Our study showed that when the occurrence of an event takes into account a competing risk, and in this case, it was death, SHR showed that APIs and Black individuals as a more disadvantaged group than Whites. The reasons for this are not completely clear, but one hypothesis is that API and Black individuals have higher mortality following a bladder cancer diagnosis compared to Whites. The idea behind this is that the in our sub-distribution hazard function, we took into consideration people who did not experience a second primary cancer but have experienced the competing event (death). These people who died are now included in the risk set as having a kind of immortal time to develop primary cancer (48). And when looking at the statistics, we find that Black patients, for example, have a higher cancer-related death compared to Whites by 70% after suffering from bladder cancer (49). However, we need to keep in mind that in our study of these two groups, the 95% CI: of SHR was very close to the inclusion of the value of 1 (0.87–0.96), which can render the SHR of 0.91 in Black individuals not that highly statistically significant and that it might be prone to changes with different sample sizes.

The strengths of this study include a large sample size from a more recent period and a long-term follow-up, in addition to implementing a competing risk analysis that accounts for competing events such as mortality which is particularly important for patients with bladder cancer who often belong to an old generation. Our study has significant limitations that warrant consideration. To begin, key critical data, such as comorbidities and treatment toxicity in patients with previous cancer, were missing from the SEER database. Second, our analysis is constrained by the inherent shortcomings of retrospective databases, which are prone to selection bias, and some results, although having a low *p*-value, their upper or lower confidence limit was closer to 1, so caution is needed when interpreting the data for clinical use. In addition, several limitations can arise from the very estimation of ethnic groups—we cannot address the factors of comorbidities and confounding factors for cancer incurrence; socio-demographic issues are also not accurately portrayed by ethnicity designation alone. Furthermore, the database used is from the United States, and great care should be taken when extrapolating the findings to these different races in other nations or locations. All of the findings in our study, we believe, should be prospectively verified. And to better comprehend the differences in SPMs between groups and the healthcare inequities, a detailed conversation of the biology of race, how race is entwined with geographical, social, and cultural conceptions, and its incorporation into clinical practice is necessary.

## Conclusion

It is the first-time large-scale data collection has been used to investigate the impact of race and histological variant in developing a second primary cancer in patients with bladder cancer. We also ended up finding that being from a specific race would influence both the risk of having an SPM and what type of malignancy it would be. This study might be useful in determining tailored therapeutic therapy and adjusting follow-up techniques. Because of the limitations of our investigation, further focused and prospective studies are needed to confirm our findings. But it seems reasonable to infer that API and Black individuals surviving bladder cancer should be more cautious than White patients when it comes to SPMs.

## Data availability statement

The original contributions presented in the study are included in the article/Supplementary material, further inquiries can be directed to the corresponding author/s.

## Author contributions

BF, CZ, and XZ conceived and supervised the study. BO, ZY, and JC contributed to data collection and assembly. All authors performed data analysis, interpretation, contributed to writing the manuscript, reviewed, and approved the final manuscript.

## Funding

This work was supported by the National Natural Science Foundation of China (81873626, 81902592, and 82070785), Hunan Natural Science Foundation (2020JJ5884 and 2020JJ5916), and Hunan Province Young Talents Program (2021RC3027).

## Conflict of interest

The authors declare that the research was conducted in the absence of any commercial or financial relationships that could be construed as a potential conflict of interest.

## Publisher's note

All claims expressed in this article are solely those of the authors and do not necessarily represent those of their affiliated organizations, or those of the publisher, the editors and the reviewers. Any product that may be evaluated in this article, or claim that may be made by its manufacturer, is not guaranteed or endorsed by the publisher.
